# Increasing the capacity of policy agencies to use research findings: a stepped-wedge trial

**DOI:** 10.1186/s12961-018-0408-8

**Published:** 2019-02-06

**Authors:** Anna Williamson, Daniel Barker, Sally Green, Catherine D’Este, Huw T. O. Davies, Louisa Jorm, Anthony Shakeshaft, Sian Rudge, Sally Redman

**Affiliations:** 1 The Sax Institute, PO Box K617, Haymarket, Sydney, NSW 1240 Australia; 2 The University of Sydney, Sydney, Australia; 3 The University of New South Wales, Sydney, Australia; 4 The University of Newcastle, Newcastle, Australia; 5 Australasian Cochrane Centre, Melbourne, Australia; 6 Monash University, Melbourne, Australia; 7 Australian National University, Canberra, Australia; 8 University of St Andrews, St Andrews, Scotland

**Keywords:** Policy, evidence, knowledge translation, knowledge mobilisation, intervention

## Abstract

**Background:**

This paper describes the trial of a novel intervention, Supporting Policy In health with evidence from Research: an Intervention Trial (SPIRIT). It examines (1) the feasibility of delivering this kind of programme in practice; (2) its acceptability to participants; (3) the impact of the programme on the capacity of policy agencies to engage with research; and (4) the engagement with and use of research by policy agencies.

**Methods:**

SPIRIT was a multifaceted, highly tailored, stepped-wedge, cluster-randomised, trial involving six health policy agencies in Sydney, Australia. Agencies were randomly allocated to one of three start dates to receive the 1-year intervention programme. SPIRIT included audit, feedback and goal setting; a leadership programme; staff training; the opportunity to test systems to facilitate research use in policies; and exchange with researchers. Outcome measures were collected at each agency every 6 months for 30 months.

**Results:**

Participation in SPIRIT was associated with significant increases in research use capacity at staff and agency levels. Staff reported increased confidence in research use skills, and agency leaders reported more extensive systems and structures in place to support research use. Self-report data suggested there was also an increase in tactical research use among agency staff. Given the relatively small numbers of participating agencies and the complexity of their contexts, findings suggest it is possible to effect change in the way policy agencies approach the use of research. This is supported by the responses on the other trial measures; while these were not statistically significant, on 18 of the 20 different measures used, the changes observed were consistent with the hypothesised intervention effect (that is, positive impacts).

**Conclusions:**

As an early test of an innovative approach, SPIRIT has demonstrated that it is possible to increase research engagement and use in policy agencies. While more work is needed to establish the replicability and generalisability of these findings, this trial suggests that building staff skills and organisational structures may be effective in increasing evidence use.

**Electronic supplementary material:**

The online version of this article (10.1186/s12961-018-0408-8) contains supplementary material, which is available to authorized users.

## Introduction

There is widespread agreement that research can make a useful contribution to health policy development [1–3], yet many opportunities to use evidence in policy are currently missed. In recognition of this, governments internationally have pledged to increase their use of evidence in the development of health policies, programmes and services [4–7].

Research evidence will only ever be one of many factors considered in developing policies and programmes [8–10]. Moreover, research use is increasingly regarded as a social, interactive, highly contingent and context-dependent process [11], and policy agencies can be seen as complex organisations embedded in an equally complex external environment. While perspectives from political science have highlighted the importance of political and institutional factors [12], in many cases, these aspects offer few avenues for influence by either research actors or policy agencies, and there remains a need to better understand the local capacities within policy-making agencies.

Policy-makers manage diverse stakeholders, including government and opposition politicians, community and professional advocacy groups, and the media; they juggle competing priorities and tight timelines, and are obliged to make decisions even when evidence is lacking and where the costs and benefits of different options are ambiguous [13, 14]. Much of this work focuses on generating creative and robust solutions to what are often wicked problems (i.e. multifactorial interdependent social concerns for which there is no agreed evidence base or solution) [15]. There are high levels of political and media scrutiny, multiple legislative and compliance frameworks, and limited resources. In short, policy work is “*…embedded in intricate networks of physical, biological, ecological, technical, economic, social, political, and other relationships*” ([16], p. 505). Despite this complexity, however, there are real opportunities to enhance the role that research plays in decision-making [8].

There is increasing appetite and willingness to use evidence from research in policy and a corresponding interest in building the internal capacity of policy agencies to do so effectively within their complex environment (e.g. [17, 18]). Interventions to support more (and more effective) research use can be designed and tested drawing on the evidence base to date of what helps and what hinders effective use. Effective capacity-building programmes will likely focus not just on individual attitudes and behaviours, but also on the social and organisational context and on the structures, processes and environments that surround policy workers. Policy agencies often have strikingly different cultures, resources and remits [19, 20] and they value and use research evidence in different ways and to varying extents [21–23]. Effective capacity development programmes will likely need to take into account the complexity and diversity of policy agencies; programmes should also be sufficiently intense to be capable of resulting in significant change while being acceptable in terms of resource and time demands within this time-pressured environment. Capacity-building programmes will also need to be multi-level and multi-faceted to support change at different levels of the organisation simultaneously – increasing the skills of staff in finding research, for example, is unlikely on its own to be effective if the prevailing organisational culture does not value research.

Given the growing number of programmes designed to build the capacity of policy agencies to use research, it is important to understand what works in practice. Using an inclusive definition of capacity-building, a recent review [24] identified 22 studies which described an evaluation of potentially relevant strategies to increase the use of research amongst administrative policy-makers in policy agencies published between 2001 and 2016 – 12 of these studies have been published since 2013. These studies examined a range of strategies designed to prompt improvements in diverse areas, such as access to research findings (e.g. [25]), skills (e.g. [26]), systems (e.g. [27]) or interaction with researchers (e.g. [28]). The interventions being evaluated fell along a continuum of complexity, with most studies examining relatively simple strategies such as the provision of an evidence brief. While many interventions addressed more than one aspect of capacity [23, 29], relatively few were complex, or targeted at multiple levels within the organisational structure or were designed to change culture or organisational factors (e.g. [23, 30]). For example, Pierson et al. [30] evaluated one public health organisation’s programme to build organisation and staff capacity for evidence-informed decision-making that included, among other things, training and skills enhancement, tools for literature reviews, forums for sharing knowledge, restructuring of the library and creation of a specialist position. In another example, Waqa et al. [31] examined the impact of a project to support evidence-informed decision-making in relation to obesity in Fiji; the intervention included mapping policy environments, analysing organisational capacity and support for evidence-informed policy-making, increasing staff skills, and facilitating evidence-informed policy briefs.

Perhaps not surprisingly, few of these studies used experimental designs; many used simple pre–post designs without a control group, or were observational studies of a new programme being implemented, often by the policy agencies themselves. Only three studies included any kind of control group and randomised to control and intervention. These three studies evaluated the use of specially designed evidence briefs [32], the extent to which policy-makers used policy-relevant systematic reviews provided by the research team [33], and the extent to which policy-makers were more likely to use a research report if they were involved in its production [28]. While these studies make important contributions to understanding what might work to improve the use of evidence, the evaluated interventions are not multi-level and attempt to modify one process rather than seeking to modify the capacities and culture of the organisation. It is perhaps to be expected that it has been more feasible to implement experimental evaluations of simple rather than complex strategies.

This paper describes a long-term programme of work designed to build the capacity of policy agencies around research use, recognising their complexity and diversity. The approach involved drawing on best evidence (in the widest sense) of what might work and then evaluating its impact, testing the assemblage in diverse policy agencies. We aimed to develop a multi-level and multi-faceted intervention capable of bringing about real change in the ways that policy organisations used research, and an evaluation that would provide dependable evidence about the value of participating in the programme. At the outset, we recognised that achieving these objectives would require the development of a substantial conceptual and methodological platform. This platform has four core components, as described below.

**Fig. 1:**
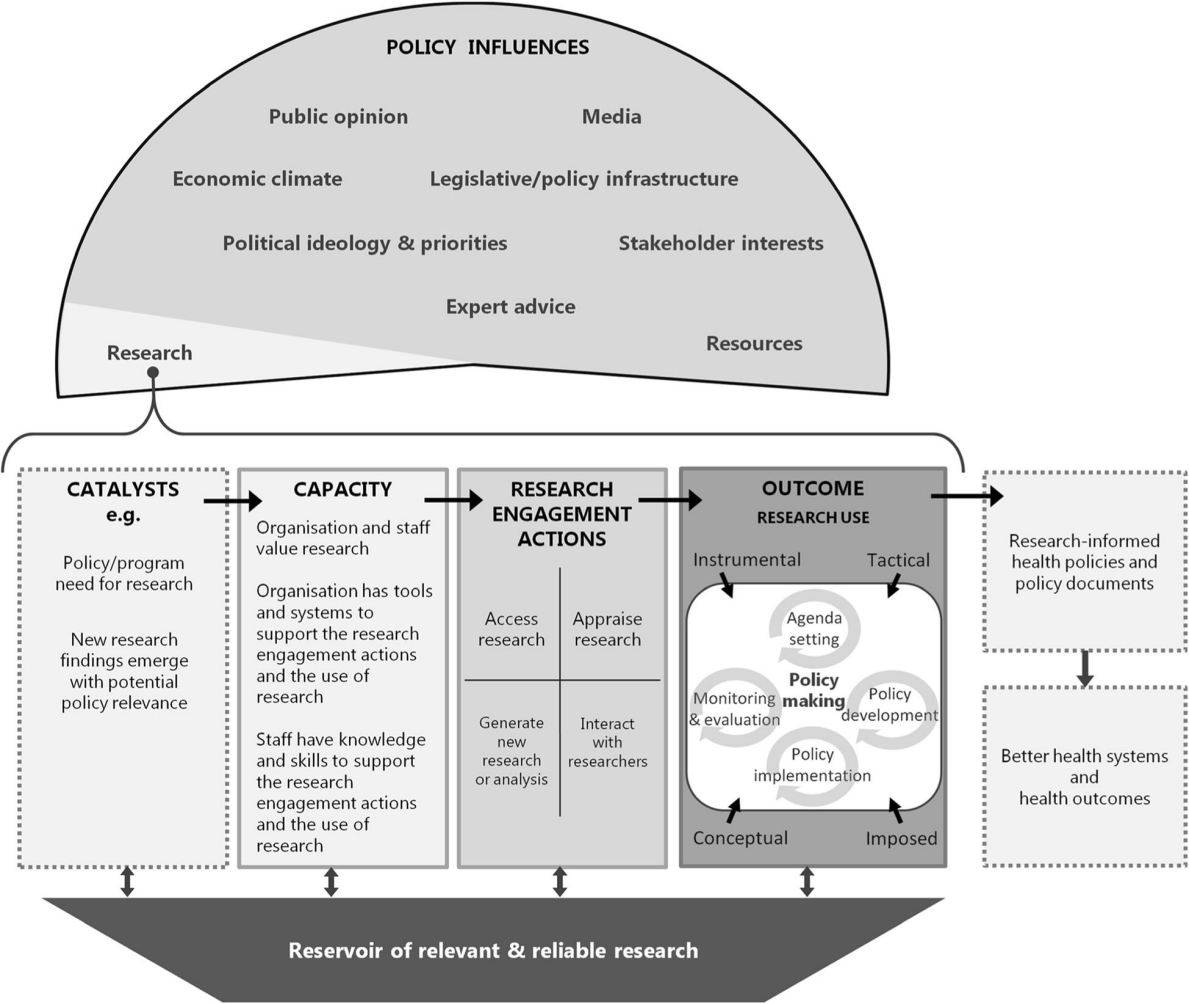
The SPIRIT action framework [35]

1. A conceptual framework to provide a guide for effective actions in each of the policy agencies (the SPIRIT Action Framework [34], described below and shown in Fig. 1);
2. Measures of outcomes that are sensitive to any changes in research use capacity in the agencies (these are described below, and summarised in Table 1);
3. An evaluative approach that balanced the need for robust evidence from careful experimentation with pragmatic considerations about feasibility;
4. A philosophy that sought to engage policy agency leaders in owning and customising the intervention while remaining true to the underlying design principles of the SPIRIT Action Framework.

**Table 1:** SPIRIT outcome measures

Outcome	Level of assessment	Tool	Data collection method	Participants
Policy-makers’ self-assessments of their research use capacity, actions and outcomes	Individual policy-maker	Seeking, Engaging with and Evaluating Research (SEER)	Online survey	All policy-makers from within participating agencies
Organisational capacity to use research as measured by the existing tools and systems to support research use	Policy agency	Organisational Research Access, Culture and Leadership (ORACLe)	Interview (face-to-face or phone) plus collection of documentation	One senior member of each policy agency, nominated by agency’s leaders
Use of research in the creation of policy documents	Policy document	Staff Assessment of enGagement with Evidence (SAGE)	Interview (face-to-face or phone) plus collection of documentation	One or two policy-makers involved in the development of the policy document being considered × 4 documents at each measurement point

First, a conceptual framework that provided a guide to action was required. The framework had to go beyond the many theories, models and frameworks that describe the research policy nexus [34], to provide a ‘field guide’ that pulls together existing understanding and insights (theoretical and empirical) to allow the design and structured testing of interventions. We developed the SPIRIT Action Framework (Fig. 1) [35] to underpin this programme of work and to form the basis for both the measures and the intervention. The SPIRIT Action Framework is based on clear and purpose-specific definitions of key concepts like ‘research evidence’ and ‘policy agencies’, and recognises that many inter-related contextual factors, of which research is only one, contribute to policy processes. The Framework reflects a hypothesis that the ‘capacity’ of an organisation to find and use research comprises the value placed on research evidence by the staff and organisation, the tools and systems to support engagement with and use of research evidence, and the knowledge and skills of staff in engaging with and using evidence. If there is sufficient capacity, and a reservoir of relevant and reliable research exists, then the agency may engage with research by accessing, appraising or generating research, or by interacting with researchers. It is hypothesised that, in turn, this research engagement will influence the use of research.

Second, we needed measures of outcome that were sensitive to changes in the capacity of the agency and able to measure variables across the SPIRIT Action Framework. These measures had to be able to capture organisational and individual level change. At the outset, we located only a few measures relevant to the capacity of agencies to find and use research and these had variable levels of psychometric testing [36–38]; none of these measures aligned well with the variables in the SPIRIT Action Framework. As part of our conceptual and methodological platform, we therefore developed and tested three new measures aligned to the SPIRIT Action Framework to measure changes in individual staff (Seeking, Engaging with and Evaluating Research (SEER)) [39]; the organisation (Organisational Research Access, Culture and Leadership (ORACLe) [40] and in the policy products produced (Staff Assessment of enGagement with Evidence (SAGE)) [41–43] (Table 1). All of these measures performed well in psychometric testing; however, it became evident that, because policy products can take over a year to develop, SAGE would not provide dependable measures of change in an intervention with short- to medium-term follow-up.

Third, we wanted to use an approach to evaluation that provided more dependable evidence about the value of participating in the programme than the simple uncontrolled pre–post test designs used by previous research. At the same time, we recognised that this would be an early test of an innovative approach and a randomised trial may be premature, as well as logistically challenging. A stepped-wedge cluster-randomised trial (CRT) design [44] has a number of advantages for testing an early stage capacity-building programme, namely that all agencies would receive the capacity-building programme; to some extent, each agency is its own control, allowing for the diversity between agencies; and the number of agencies required is potentially smaller than that required for a randomised trial, providing for a more feasible trial and a more intensive intervention [44]. While constructing our study protocol using a stepped-wedge design, we found that significant developmental work was required to establish the best approaches to sample size estimation and analysis, particularly when the number of clusters (organisations) was small. Consequently, we undertook a series of studies to investigate methods for design and analysis of stepped-wedge CRTs in these contexts [45, 46].

Finally, the intervention itself would be based on a programme logic derived from the SPIRIT Action Framework. Our programme theory held that SPIRIT would seek to engage agency leaders and motivate them to ‘own’ the intervention using audit and feedback, goal setting and programme tailoring. This approach would empower agencies to co-create a priority-focused programme incorporating locally relevant skills and knowledge and tailored to each agency’s values, goals, resources and remits. We planned that the programme would provide resources and enhance knowledge, skills and relationships through a suite of activities, tools, and opportunities to make connections across the research–policy divide. The mission of SPIRIT would be supported by agency leaders and external experts through role modelling (demonstrating or explaining how they have successfully used evidence in their work) and opinion leadership. Participating Chief Executive Officers would promote SPIRIT internally and agency liaison people would work with the SPIRIT team to facilitate the tailoring and implementation of our capacity development intervention. We envisaged that these strategies would combine to engage and resource participants at all levels of the participating organisations leading to changes in values, behaviours and agency processes. In this way, we hypothesised that SPIRIT would increase the use of research in policy processes [47].

This paper describes the trial of the SPIRIT programme in six policy agencies. It examines (1) the feasibility of delivering this kind of programme in practice; (2) its acceptability to participants; (3) the impact of the programme on the capacity of policy agencies to engage with research; and, hence, (4) the engagement with and use of research by policy agencies.

## Methods

### Design

**Fig. 2:**
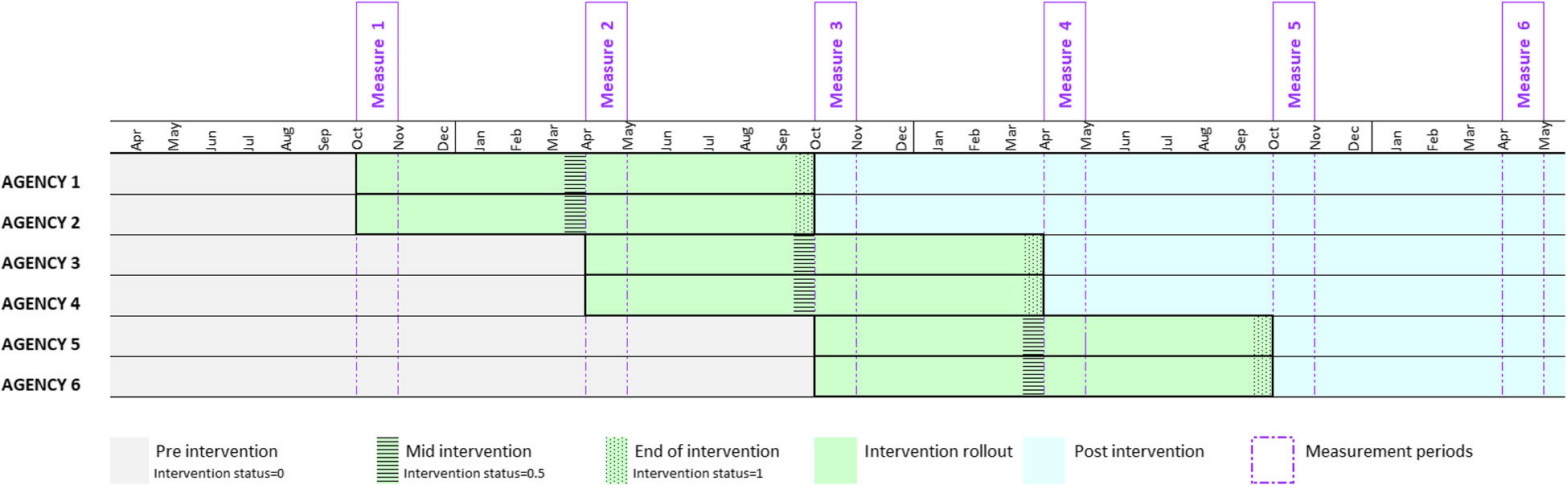
The SPIRIT stepped-wedge design

SPIRIT used a stepped-wedge CRT design with six agencies, wherein two agencies were randomly assigned to start the intervention in the first 6 months, two in the second 6 months, and two in the third 6 months. The intervention period was 12 months. Outcome measures were collected at the same time in all sites, with six measurement collection periods, 6 months apart, as shown in Fig. 2. SPIRIT ran from October 2012 until December 2015. Further details of the study design have been previously described [48].

### Participants

Agencies were eligible to participate if a significant proportion of their work was in health policy or programme development; 20 or more staff members were involved in policy or programme design, development or evaluation; and they were located in Sydney, Australia (for ease of programme provision). A total of 75 potentially eligible agencies were initially identified using government websites, 16 of these had sufficient numbers of relevant staff to be eligible. Eligible agencies were ranked based on staff numbers and level of focus on health and were approached to participate in the ranked order.

### Intervention

The SPIRIT intervention was developed as described herein. Firstly, a programme logic was developed from the SPIRIT Action Framework [35]. Secondly, a detailed literature review was undertaken to identify strategies likely to be effective in increasing the use of research in policy, which led us to draw heavily on cognitive behavioural theory [49, 50], system science [51, 52], organisational change theory [53–56] and adult learning theories [57, 58]. Although not available at the time of the intervention design, many of the principles incorporated were broadly consistent with the insights emerging from the most recent and comprehensive review [59]. The information derived from the literature review was supplemented by a review of the websites of knowledge exchange and policy agencies. Thirdly, an iterative process was used to select programme components for inclusion drawing on the literature, the knowledge exchange and policy experience of the research team and beyond, and a small formative interview study of policy-makers [60]. Finally, some components were pilot tested in a non-participating agency and the programme was refined accordingly [48].

**Fig. 3:**
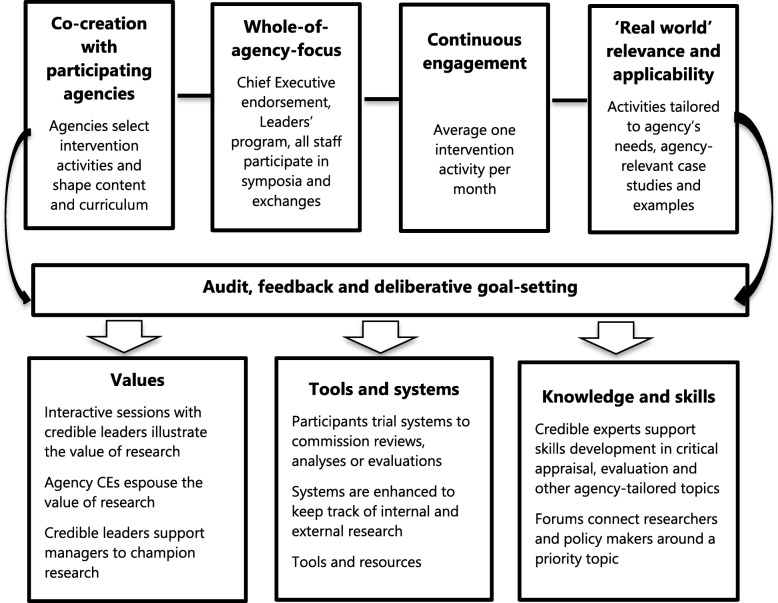
The SPIRIT intervention mapped to the four design principles

As shown in Fig. 3, four key principles underpinned the design and implementation of the SPIRIT intervention. Firstly, in order to engage agencies and increase their sense of ownership of the intervention, the intervention programme was co-created. The focus of the majority of intervention activities was determined by the agencies and all activities were tailored. Secondly, in order to promote organisational change, the intervention was designed to work at all levels of the agency, including both intervention activities for all staff and others aimed specifically at agency leaders. Next, we sought to keep the use of evidence as a priority by engaging continuously with agencies. In delivering one intervention activity per month, we aimed to strike a balance between maintaining momentum and overburdening busy agencies. Lastly, we sought to ensure that all intervention activities had ‘real world’ relevance and applicability, so they might promote real change and also be considered valuable enough to take the time to attend.

In order to facilitate implementation, the SPIRIT team assigned an individual with extensive knowledge brokering experience (experience working in the nexus between research and policy) to act as the SPIRIT Officer for each agency. The SPIRIT Officer worked closely with the internal member of staff nominated by each agency to assist with the implementation of SPIRIT in their site (Agency Liaison Person) [61] to tailor the intervention to their agency’s interests and needs.

Table 2 shows how the SPIRIT intervention was implemented in practice. As shown, the intervention began with an audit, feedback and goal-setting session with an agency-nominated leaders group. Here, agency leaders were presented with data obtained from their most recent round of measures. The facilitator (author SR) highlighted the agency’s current strengths and opportunities for improvement in terms of staff skills and confidence, the tools and systems available to support research use, and staff perceptions of leaders’ support for using research. Leaders discussed what these findings meant for their agency and used them as a jumping off point for determining what their agency’s goals for participation in SPIRIT would be and how to tailor their intervention activities.

**Table 2:** How the SPIRIT intervention was implemented in practice

Agency A	Agency B	Agency C	Agency D	Agency E	Agency F
*Data showed:* - 46% confident in their ability to appraise research evidence - 29% confident in their ability to evaluate policies or programmes	*Data showed:* - 58% confident in their ability to appraise research evidence - 42% confident in their ability to evaluate policies or programmes	*Data showed:* - 20% thought agency had well-developed relationships with external researchers, 40% felt confident in partnering with researchers - 20% confident in their ability to evaluate policies or programmes	*Data showed:* - 35% confident in their ability to access research - 31% confident in their ability to appraise research evidence - 15% confident in their ability to evaluate policies or programmes	*Data showed:* - 19% believe X has well-developed processes for programme development that provide guidance on research use - 56% confident in their ability to access research, 39% confident in their ability to appraise research evidence - 35% confident in their ability to evaluate policies or programmes	*Data showed:* - 47% confident in their ability to access research, 32% in ability to appraise research evidence - 11% confident in their ability to commission research - 42% confident in their ability to interpret the results of research, 53% confident in their ability to apply research in policy and programme work - 37% confident in their ability to evaluate policies or programmes
*Goals selected as:* - Increase appraisal capacity - Strengthen capacity to support programme evaluation - Develop capacity for some staff in the use of research findings in policy work	*Goals selected as:* - Develop capacity in appraising the quality and relevance of research - Supporting programme evaluation - Skills development in less experienced staff	*Goals selected as:* - Increase agency capacity to work with external researchers - Supporting programme evaluation, e.g. what additional information would increase the usefulness of evaluations?	*Goals selected as:* - Increase staff capacity in accessing research - Increase appraisal capacity - Increase staff capacity in supporting evaluation of policies and programmes	*Goals selected as:* - Embedding use of research into X’s systems and processes, to ensure its sustainability - Improve staff capacity to access and appraise research - Support staff capacity in programme evaluation	*Goals selected as:* Enhance skills amongst some staff in: - Accessing and appraising research - Commissioning research - Applying research in practice - Evaluating policies and programmes
Value					
Leaders forums with previous head of Australian Public Service and with international leader in capacity					
All staff symposia on the value of research evidence in policy					
Email from Chief Executive or other agency leader highlighting value of research × 4					
Skill development					
*Skill development sessions on (1 topic fixed, 2 selected by agency):* Appraisal of research evidence; evaluation (foci varied between agencies, key topics selected included overview of basic and advanced concepts; process evaluation and the use of routinely collected data; embedding evaluation in programme rollout, scaling and communication in evaluation); assessing and appraising systematic reviews; introduction to research					
*Practice exchanging with researchers on (3 selected per agency):* Clinical network; screening; social media messaging; smoke-free policies in XXX; using broader (‘soft’) practice evidence; engaging with health professionals; implementing sustainable change					
*Tested a system for* analysing locally relevant data OR commissioning an evaluation framework OR rapid review of evidence on a policy-relevant topic					

All agencies received interventions (leaders’ fora, all-staff symposium and quarterly emails from agency leaders) designed to promote the value of research evidence in policy. These were tailored to reflect the interests of each agency and what the baseline data reflected about their current evidence use culture and practices. Agency liaison people worked with their SPIRIT Officer to co-create the skills development aspects of the SPIRIT intervention for their agency, choosing both the focus area and the specific learning objectives of each activity. The majority of agencies selected skill development sessions focussing on areas that emerged as key opportunities for improvement in their audit and feedback sessions. While there was substantial cross-over in the skills development session topics chosen, the co-creation of learning objectives meant that the actual content of, for example, a session on evaluation, tended to differ substantially between agencies.

We used our networks to identify and approach the leading national and international researchers and policy experts in each relevant area of knowledge (e.g. data linkage, evaluation) and invited them to facilitate relevant activities selected by agencies. Our invited experts were asked to address the core learning objectives specified by the agency and to adhere to the high-level principles which underpinned all SPIRIT intervention activities (e.g. that they be engaging, interactive, relevant to the agency’s work, and that respect be shown for the existing skills and expertise of agency staff) but no attempt was made to force a standard delivery of sessions.

### Measures

#### Feasibility

We examined the extent to which each component of the intervention was delivered as planned. All workshops were audio recorded and field notes were written immediately afterwards. We assessed participation in the workshops and the extent to which each pre-specified core component, agency-determined learning objective (content) and the aforementioned SPIRIT principle (interaction) were delivered using a four-point descriptive scale of extensive/moderate/limited/not at all. [62]. Delivery of the non-workshop elements of the intervention were measured as follows: (1) Chief Executive emails – recording whether each planned email was sent, and by who within the organisation, and (2) systems testing – recording whether the final product requested (a rapid evidence review, an evaluation framework or an analysis of locally relevant data) was delivered to and signed off by each agency.

#### Acceptability

Acceptability was measured using participant feedback forms. These forms elicited yes/no ratings on six statements, as follows: (1) the workshop was interesting, (2) the workshop was relevant to my work, (3) the workshop was realistic about the challenges and constraints of our work, (4) the presenter had appropriate knowledge and skills, (5) it is likely that I will use information from this workshop in my work, (6) it is likely that SPIRIT will benefit my agency. Open-ended responses were sought in regard to three questions, namely ‘What worked well?’, ‘What could be improved?’, and ‘Any other comments?’. Feedback forms were collected immediately after each workshop.

#### Impact on research use capacity

1. Staff capacity to use research was measured using the online survey SEER [21] and assessed the extent to which staff valued research (7 items, score range 7–35), were confident in their ability to access, appraise and generate research, interact with researchers and use research (7 items, score range 7–35), felt their organisation valued research use (5 items, score range 5–25), and felt that their organisation has the tools and systems required to support research use (7 items, score range 7–21).
  Agency staff were eligible to complete SPIRIT measures if they wrote health policy documents or developed health programmes, or made or contributed significantly to policy decisions about health services, programmes or resourcing; they were over 18 years of age; and they consented to participate in the study.
2. Organisation capacity to use research (availability of relevant systems and tools): We conducted semi-structured qualitative interviews with one senior staff member from each agency to assess the extent to which the agency had in place tools and systems to encourage the use of research (ORACLe) [40]. Twenty three questions covered the following seven dimensions: (1) processes that encourage or require the examination of research in policy and programme development; (2) tools and programmes to assist leaders of the organisation to actively support the use of research in policy and programme development; (3) strategies to provide staff with training in using evidence from research in policy and in maintaining these skills; (4) organisational strategies to help staff to access existing research findings; (5) methods to generate new research evidence to inform the organisation’s work; (6) methods to ensure adequate evaluations of the organisation’s policies and programmes; and (7) strategies to strengthen research relationships. Two researchers separately scored the responses of each participant on a 1–3 scale (no; some or limited; yes, very much so). Inter-rater reliability was high (95%). Scores were calculated for each domain and a total score was obtained based on a previously described method that accords with the views of leaders in policy and knowledge exchange about key systems and tools [63].

#### Impact on research engagement

We also used SEER to assess changes in research engagement. Specifically, we examined the extent to which staff reported engaging with research over the past 6 months by accessing research (4 items, score range 0–3), appraising the quality and relevance of research (3 items, score range 0–3) and/or generating research (3 items, score range 0–1), and by interacting with researchers (6 items, score range 6–24).

#### Impact on research use

Extent of use of research in policy development was measured using four items from the SEER online survey, with scores ranging from 1 to 6. One additional question assessed whether each of the four types of research use was undertaken, namely conceptual (where research is used to understand an issue); instrumental (where research is used to develop specific policy content); tactical (where research is used to persuade others); and imposed (where research is used to meet organisational requirements). The score range for each of these items was 0–1.

### Ethics

Ethics approval was granted for the overall CIPHER programme of work by the University of Western Sydney Human Research and Ethics Committee (HREC Approval H9870). No harms of participation were identified for either individual participants or participating agencies.

### Analysis

#### Feasibility and acceptability

The number and percentage of individuals participating in each intervention activity was obtained as a measure of the planned intervention activities implemented. Direct observation of sessions was used to determine the extent to which each pre-determined key component of session content and style were present. The number and percentage of participants in each session responding ‘yes’ to each of six statements measuring aspects of acceptability was calculated for each agency, combining data from the four different types of workshops (symposia, research exchanges, leaders’ forums, and audit feedback forums).

#### Capacity and research engagement and use

Statistical analysis was undertaken by statisticians blind to identity of the agencies using SAS version 9.4. Summary statistics were presented as means and standard deviations for dimension scores and frequencies and percentages for binary outcomes, for the pre-intervention, mid-intervention and end of intervention periods.

The unit of analysis was the individual for SEER and the agency for the ORACLe outcome measure. Analysis was undertaken using linear regression for domain scores and logistic regression for binary outcomes, and including intervention phase (pre, mid, post intervention) and measurement time as covariates. Both types of regression models were fitted within a generalised linear mixed model framework to adjust for the correlation of measures within individuals and within agency for SEER and within agency for ORACLe. Using a 5% significance level, the study had at least 80% power to detect an average difference in SEER scores of 1.5, assuming a standard deviation of 6 [63]. Additional information on the statistical methods used can be found in the technical appendix (Additional file 1).

## Results

### Participants

All six agencies invited to participate in the intervention agreed to do so. Five were state based and one was a national organisation. Three of the participating agencies conducted work focussed on specific areas of health or healthcare, while three worked across public health and health systems improvement. All had been operating for at least 3 years but most were subject to recent or current restructures.

As shown in Table 3, agencies differed substantially at baseline in relation to core aspects of their work, such as remit and geographic location, and practical factors related to the skills mix and even location of their staff. Agency culture around evidence use appeared to differ substantially at baseline, such that, for example, while almost all the staff at some agencies reported that evaluation of their policies or programmes was expected, this was a minority view in other organisations. Likewise, almost all staff at some agencies felt they were encouraged to interact with researchers, yet this was not the case in others. In keeping with this, agencies differed in the extent to which they already had well-established systems and structures to support the use of evidence. All had a different combination of pre-existing relevant systems and structures, and all had capacity to improve in this area.

**Table 3:** Differences between SPIRIT agencies

	Agency A	Agency B	Agency C	Agency D	Agency E	Agency F
Geographic focus of work	New South Wales	Australia	New South Wales	New South Wales	New South Wales	New South Wales
Remit	Public health	Health systems improvement	Health systems improvement	Specific aspect of health	Health systems improvement	Specific aspect of health
Staff location	Single building	Single building	Single building	Single building	Single building	Various locations throughout NSW
Staff composition	Primarily career public servants	Mix of clinicians and public servants	Mix of clinicians and public servants	Mix of clinicians and public servants	Mix of clinicians and public servants	Primarily clinicians, some public servants
Aspects of agency evidence use culture						
It is usually or always expected that policies or programmes be evaluated Percentage of staff within each agency responding ‘yes’: range 34–93%						
Interaction with researchers or research organisations is usually or always encouraged Percentage of staff within each agency responding ‘yes’: range 25–96%						
Agency systems and structures to support the use of research evidence						
Do your policies on how to develop policies or programmes encourage or require research use? Range of agency leaders’ responses: ‘no’ to ‘yes, very much so’						
Does your organisation provide training for staff in how to access, appraise and apply research? Range of agency leaders’ responses: ‘no’ to ‘yes, very much so’						

### Feasibility

Despite its intensity and complexity, SPIRIT was implemented as planned; all agencies participated in the entire 30-month study and all 14 planned intervention activities were delivered in each agency within a 10–17 month period. The degree to which the agreed core components of each session were delivered varied, but was generally high – each aspect of planned core content (to address each pre-specified learning objective) was delivered to a moderate or extensive degree in all 52 intervention workshops. In 83% of the workshops, the defined delivery style was used.

### Acceptability

On average, eligible participants attended 3–4 workshops and these were highly acceptable, as shown in Table 4. Free response data from participant feedback forms suggested that participants highly valued the way in which workshops helped them link ideas to practice, used real-world examples and imparted ‘practical, take-home stuff’. The high calibre of the presenters was frequently commented upon in terms of their reputation, experience and expertise, and their passion for the topic. Presenters who had real world experience were particularly valued.

**Table 4:** Feedback form responses for intervention workshops across all agencies

Feedback form statement (Yes/No responses)	Yes (numerator/denominator))	Yes (%)
1. The workshop was interesting	491/501	98
2. The workshop was relevant to my work	472/503	94
3. The workshop was realistic about the challenges and constraints of our work	262/280	94
4. The presenter had appropriate knowledge and skills	535/542	99
5. It is likely that I will use information from this workshop in my work	325/341	95
6. It is likely that SPIRIT will benefit my agency	280/285	98

### Impact

The number of participants nominated by the Agency Liaison People as eligible to complete SEER ranged from 20 to 79 per agency over the six measurement rounds. The overall response rate for SEER was 56%. The number of participants per agency for any measurement ranged from 11 to 40 (Table 5). One senior member of staff from each agency completed the ORACLe interview at each time point.

**Table 5:** Participation in the SPIRIT measures across the six measurement periods

	Measure 1 mean (min, max)	Measure 2 mean (min, max)	Measure 3 mean (min, max)	Measure 4 mean (min, max)	Measure 5 mean (min, max)	Measure 6 mean (min, max)
SEER number of respondents	25.7 (16, 36)	17.3 (11, 28)	20.8 (14, 32)	23.5 (18, 40)	21.0 (17, 27)	20.8 (18, 26)
SEER response rate (%)	63%	38%	50%	58%	58%	57%

#### Impact of SPIRIT on capacity to use research

Tables 6 and 7 show the summary statistics and intervention effect estimates in relation to capacity to use research. For SEER data, the intervention effect estimates are the amount an agency’s score increases after receiving the SPIRIT intervention programme compared to what would have been expected in the absence of an intervention. Negative values represent an average decrease in outcome score following the intervention.

**Table 6:** Impact of SPIRIT on research use capacity (individual level): SEER self-report survey summary statistics for pre-intervention, during roll out and upon receipt of full intervention, and intervention effect estimates with 95% confidence intervals (CIs)

Outcome	Pre-intervention (*n* = 265) mean (SD)	Intervention roll out (*n* = 254) mean (SD)	Full intervention received (*n* = 256) mean (SD)	Intervention effect (95% CI)	*p* value
Value individual places on using research (maximum = 35)	28.81 (3.91)	28.76 (4.22)	28.93 (3.94)	0.05 (− 1.5 to 1.61)	0.9491
Confidence in using research (maximum = 35)	23.07 (6.08)	24.40 (5.73)	24.63 (5.61)	1.58 (0.12 to 3.05)	0.0342
Value the organisation places on using research (maximum = 25)	19.32 (3.64)	19.60 (3.38)	20.30 (3.07)	0.45 (− 0.78 to 1.68)	0.4721
Tools and systems organisation have to support research use (maximum = 21)	17.85 (5.05)	18.09 (4.23)	18.70 (3.98)	0.56 (− 1.28 to 2.4)	0.5511

Results from generalised linear mixed model adjusted for correlation of observations within agency, and within individuals, and including time as a covariate; *p* value for likelihood ratio test

**Table 7:** Impact of SPIRIT on research use capacity (agency level): estimated intervention effect, 95% confidence interval (CI) and *p* value for each of the seven ORACLe domains and the overall score^a^

Domain	Intervention Effect (95% CI)	*p*-value
Domain 1: Documented processes to develop policies that encourage or mandate the use of research (maximum score = 3)	− 0.23 (− 1.15 to 0.69)	0.6112
Domain 2: Tools and programmes to assist leaders of the organisation to actively support the use of research in policy and programme development (maximum score = 3)	0.01 (− 0.52 to 0.54)	0.9778
Domain 3: Availability of programmes to provide staff with training in using evidence from research in policy and in maintaining these skills (maximum score = 3)	1.28 (0.5 to 2.05)	0.0022
Domain 4: Availability of supports and tools to help staff access and apply research findings (maximum score = 3)	0.5 (− 0.13 to 1.14)	0.1150
Domain 5: Presence of systems/methods to generate new research evidence to inform the organisation’s work (maximum score = 3)	0.76 (− 0.14 to 1.65)	0.0946
Domain 6: Clear methods to allow adequate, evidence-informed evaluations of the organisations’ policies and programmes (maximum score = 3)	0.37 (− 0.54 to 1.29)	0.4105
Domain 7: Mechanisms that help strengthen staff relationships with researchers	0.57 (0.15 to 0.99)	0.0100
Total ORACLe Score	2.18 (0.21 to 4.14)	0.0314

^a^6 observations per agency for 6 agencies

Results from generalised linear mixed model adjusted for correlation of observations within agency and including time as a covariate; *p* value for likelihood ratio test

### Staff research use capacity

We observed a significant improvement in the extent to which participants reported confidence in their research use skills associated with the intervention (*p* = 0.03). For the other self-reported measures of research use capacity, changes were in the expected direction but did not reach statistical significance.

### Organisational level research use capacity (availability of relevant systems and tools)

Our data show a significant overall increase in the extent to which agencies had the tools and systems to support engagement with and use of research evidence associated with the intervention (Total ORACLe Score, *p* = 0.03). Significant improvements were also noted, specifically in relation to the availability of programmes to provide staff with training in using evidence from research in policy and in maintaining these skills (*p* = 0.002) (SPIRIT sessions were not counted as research use training provided by the agency and thus do not count towards this significant result) and mechanisms that help strengthen staff relationships with researchers (*p* = 0.01). There was also some evidence of improvement in relation to presence of systems/methods to generate new research evidence to inform the organisation’s work; however, this was only statistically significant at the 10% level (*p* = 0.095). Again, looking across the seven domains, we can see that in all except one of these, the changes observed were in the expected direction, although they mostly lacked statistical significance.

#### Impact of SPIRIT on research engagement

The proportion of people who reported accessing primary research increased, although this was significant at the 10% level only (*p* = 0.098, Table 8). No other significant changes in research engagement were observed, although again four of the five outcomes recorded changes in the expected direction.

**Table 8:** Impact of SPIRIT on research engagement: SEER self-report survey summary statistics for pre-intervention, during roll out and upon receipt of full intervention, and intervention effect estimates with 95% confidence intervals (CIs)

Outcome	Pre-intervention (*n* = 265)	Intervention roll out (*n* = 254)	Full intervention received (*n* = 256)	Intervention effect (95% CI)	*p* value
	mean (SD)	mean (SD)	mean (SD)		
Accessed synthesised research (maximum = 2)	0.85 (0.70)	0.98 (0.72)	0.97 (0.66)	0.14 (− 0.18 to 0.46)	0.3988
Accessed primary research (maximum = 2)	1.29 (0.79)	1.51 (0.69)	1.53 (0.71)	0.27 (− 0.05 to 0.59)	0.0986
Appraised research (maximum = 3)	1.74 (1.21)	2.43 (0.95)	2.55 (0.83)	0.07 (− 0.43 to 0.57)	0.7817
Generated research (maximum = 3)	1.25 (1.05)	1.56 (1.01)	1.57 (1.01)	0.12 (− 0.3 to 0.54)	0.5761
Interacted with researchers (maximum = 24)	12.44 (4.70)	12.67 (4.56)	12.58 (4.80)	− 0.08 (− 1.7 to 1.54)	0.9233

Results from generalised linear mixed model adjusted for correlation of observations within agency and within individuals, including time as a covariate; *p* value for likelihood ratio test

#### Impact of SPIRIT on research use

There was no overall increase in research use based on SEER data (Table 9). The intervention is estimated to have significantly increased the odds of tactical research use (using research to persuade) (OR 5.03, 95% CI 1.46–17.39). However, the odds of reporting the three other types of research use, while greater than one in all cases (indicating increased research use across the board), did not reach statistical significance. Confidence intervals here were very wide.

**Table 9:** Impact of SPIRIT on research use: SEER self-report survey summary statistics for pre-intervention, during roll out and upon receipt of full intervention, and intervention effect estimates with 95% confidence intervals (CIs)

Outcome	Mean (SD)			Intervention Effect (95% CI)	*p* value
	Pre-intervention (*n* = 265)	Intervention roll out (*n* = 254)	Full intervention received (*n* = 256)		
	*n* (%)	*n* (%)	*n* (%)	odds ratio (95% CI)	
Conceptual research use (yes/no)	176 (0.74)	184 (0.84)	184 (0.87)	2.32 (0.49 to 11.06)	0.2892
Instrumental research use (yes/no)	183 (0.77)	199 (0.90)	188 (0.89)	0.94 (0.23 to 3.89)	0.9302
Tactical research use (yes/no)	141 (0.59)	175 (0.80)	181 (0.86)	5.03 (1.46 to 17.39)	0.0108
Imposed research use (yes/no)	57 (0.24)	87 (0.40)	95 (0.45)	1.14 (0.27 to 4.76)	0.8615

Results from generalised linear mixed model adjusted for correlation of observations within agency and within individuals, including time as a covariate; *p* value for likelihood ratio test

## Discussion

Our findings indicate that the tailored, multifactorial SPIRIT intervention could be implemented according to plan and was highly acceptable to participants. There was a significant increase in some aspects of the capacity to use research at both the staff and agency level following the intervention. After the intervention, staff at participating agencies reported significantly greater confidence in their research use skills and agency leaders reported their agency to have more extensive systems and structures in place to support research use, particularly in relation to staff training and mechanisms to strengthen relationships with researchers. An increase in tactical research use was also reported by staff. On almost all other measures, the changes observed were consistent with positive effects but failed to reach statistical significance. In some aspects (such as staff research use capacity) measures were already high pre-intervention, suggesting little room for improvement. In other areas, the shifts in scores were only limited, being of insufficient magnitude for statistical significance in the modest sample of agencies included. However, on some outcomes (such as reported research use) the changes were non-trivial (for example, a doubling in the conceptual use of research) and the confidence intervals were very wide, leaving open the possibility that the findings are consistent with sizable effects (as well as, of course, with no effect).

Overall, our view is that, looking at the findings in the round, this early test of an innovative approach demonstrates the likelihood of small but worthwhile gains, and the potential (but as yet not substantiated) for larger impacts on research use practices in this notoriously difficult area and in the face of many situational impediments [8]. Mounting a study of this size and complexity was very challenging, and considerable thought and resource will be needed to develop studies with greater power and precision.

There were considerable differences between agencies in terms of culture, evidence use skills and practices, and even expectations about how sessions should be delivered. Given this, we postulate that the highly tailored nature of the intervention was essential, not just in engaging agencies, but also in facilitating the creation of a suite of intervention activities that would be considered relevant and useful in their vastly different contexts. As reported elsewhere [61], the agency liaison people played a key role, both in supporting this tailoring process and in promoting the intervention internally [61]. Another key driver of the acceptability of the intervention activities appeared to be the calibre of the experts who led them, with many participants volunteering feedback related to this.

SPIRIT sought to increase the capacity to use research at both an individual and an agency level. We conceptualised the three 2-hour ‘skills-building’ sessions agencies received as a means to pique attendees’ interest about particular aspects of research use, rather than as being sufficient to significantly increase skills in and of themselves. Instead, we hoped they would stimulate a conversation about the use of evidence in policy that may, in combination with the other intervention activities, help spark a shift in agencies further towards an enhanced culture of research use. Indeed, while other studies of ‘public health professionals’ [64–66] have reported increased evidence-use skills and/or confidence following skill-building training, these training programmes have tended to be significantly more intensive (6 weeks training in total [64], 1 week [65], half a day session on one topic [66]) than the training provided by SPIRIT.

Consequently, we hypothesise that the increased self-reported confidence in research use skills following participation in SPIRIT is attributable to three things. First, the deliberate use of a ‘real world’ stance in each SPIRIT session appeared to be important. For example, the session on appraisal of research evidence (which was unexpectedly popular and requested by four out of six agencies) was not devoted to teaching how to thoroughly appraise the science of a primary research article, but rather the session focussed on how to tell whether a review is reliable, and how to determine the applicability of a paper/review to the agency’s work.

Second, it is likely that some of the increase in confidence in research use skills among staff participating in SPIRIT was related to the increase in systems and tools to support the use of evidence at the agency level. For example, agency leaders reported a significant increase in the provision of training in research use skills. International leaders in knowledge mobilisation agreed that the availability of programmes to provide staff with training in using evidence from research in policy, and in maintaining these skills, is very important [40]. The observed increase in mechanisms to strengthen staff relationships with researchers may also have helped to increase staff confidence in their ability to use research in their work. In addition, there was some evidence of an increase in the generation of research evidence by agencies over the study period, an activity that is also likely to build staff skills and confidence in research use.

Several strategies included in SPIRIT may have contributed to better systems and structures to support research use within the agencies. For example, the initial audit and feedback sessions generally stimulated discussion across the leadership team about opportunities to strengthen systems and structures. Similarly, agency leaders were provided with practical tools and strategies to improve systems in one of the leaders’ forums and there was discussion about how to apply these within the agency. Completing the ORACLe interview in and of itself is also likely to have given leaders space to reflect on their agency’s current strengths and opportunities for improvement in providing the systems and structures to support research use and to have given them ideas about systems to implement that they may not have otherwise had. Lastly, by facilitating intensive, structured interactions between agency staff and leading researchers around specific topics pertinent to their work, the three tailored ‘research exchanges’ may have made research seem more relevant to some policy-makers. Taken together, these results suggest that a multilevel, multifaceted intervention that includes strategies to build agency systems and tools may increase some aspects of policy-makers’ capacity to use research (especially confidence in their research use skills).

The SPIRIT Action Framework [35] predicts that increased research use capacity would lead to increased engagement with research evidence. We saw some evidence of this in the trial, with an increase in the extent to which staff reported accessing primary research; however, other aspects of reported research engagement appeared largely unchanged. The Framework further suggests that an increase in engagement would lead to an increase in research use. Again, the trial found some evidence of this, with a significant increase in the proportion of staff who reported using research to persuade others to a point of view or course of action (tactical use of research). While this is sometimes considered to be an undesirable way of using research, in the real world, it might be fundamental to bringing about change. Policy-makers must have the policies or programmes they work on approved by senior staff, and potentially government, and will also need to convince stakeholders that the proposed changes are useful and important. The increased tactical research may in fact reflect an increase in the culture of evidence use within agencies. Overall, the trial results appear to have provided some support for the SPIRIT Action Framework and for the usefulness of the four principles that underpinned the design and implementation of SPIRIT.

Further research is needed to explore whether the findings reported here are generalisable to other contexts. We were, for example, somewhat surprised at the willingness of agencies to engage in SPIRIT at the outset given the substantial time commitment required coupled with the vulnerability inherent in having the evidence use capacity of your agency assessed, both of which are potential stumbling blocks. That all six agencies which were invited to participate accepted signalled a clear appetite for increasing the use of evidence amongst policy agencies in New South Wales. This attitude was further reflected by the high levels of participation in the measures and intervention activities across the trial. It may be that this willingness was the result of the long-term relationship between the Sax Institute, which implemented the intervention, and the participating agencies. It is possible that this relation had established a high level of trust without which participation in SPIRIT may have seemed too risky and onerous a prospect to take on. While a cost benefit analysis was not feasible for the current study, future research might usefully include this. A final possible direction for future research may be exploring whether an adapted version of this intervention might be feasible, or indeed effective, amongst elected policy-makers.

In conclusion, while interventions in this space are challenging to design, implement and evaluate, using a clear platform for a structured (and evidence-informed) intervention with attention to underlying principles, alongside an appropriately balanced evaluation strategy has yielded real insights and hope that modest gains can be made through concerted effort. SPIRIT has provided a demonstration that it is possible to increase research engagement and use in policy agencies, as well as reinforcement of some of the underlying design principles of such interventions teased out from a wide range of prior studies and a tested methodological framework now ready for further evaluation work.

## Additional file

Additional file 1: Statistical methods and power. (DOCX 25.7 kb)

## Abbreviations

CRT: cluster-randomised trial
ORACLe: Organisational Research Access, Culture, and Leadership
SAGE: Staff Assessment of engagement with Evidence from research
SEER: Seeking, Engaging with and Evaluating Research
SPIRIT: Supporting Policy in Health with Research: an Intervention Trial
