# Supplementary material for: Increasing the capacity of policy agencies to use research findings: a stepped-wedge trial
Source: Health Res Policy Syst. 2019 Feb 6;17:14. doi: 10.1186/s12961-018-0408-8 (PMC6366302; doi:10.1186/s12961-018-0408-8)
Supplement: Supplementary file 1 — Statistical methods and power. (DOCX 25.7 kb) [file 12961_2018_408_MOESM1_ESM.docx]

# Technical appendix

**Statistical methods and power**

The statistical analysis was undertaken by analysts blind to the identity of the agencies. The SAS statistical package was used and summary statistics are presented as means and standard deviations for domain scores and frequencies and percentages for binary outcomes, for the pre-intervention, roll-out and post intervention periods.

The unit of analysis was the agency for the SAGE and ORACLe outcome measures and the individual for the SEER outcome measure. Analysis was undertaken using linear regression for domain scores and logistic regression for binary outcomes within a generalised linear mixed models (GLLMs) framework to adjust for correlation of measures within agency for SAGE and ORACLe, and within agencies and individuals for SEER measures. Intervention status was classified as 0 when the agency was in the control condition, 0.5 during the roll-out period and 1 post intervention (at the end of the 12 month implementation period). In each model, the intervention effect was estimated as the difference between the post intervention and pre-intervention levels of the outcome after adjusting for time as a fixed effect. The Likelihood Ratio Test was used to assess the significance of the intervention effect, and a 5% significance level used.

A GLMM was used instead of a generalised linear model with fixed effect for agency as specified in the study protocol since recent methodological work by our team has demonstrated that, with six clusters, this GLMM model has less bias on average than the GLM with fixed effect for agency while maintaining the nominal type I error rate of 5%[[19](#_ENREF_19)]. We used the sample size method proposed by Hooper et al to undertake post hoc power calculations, updating our a priori estimates based on actual study design and parameter estimates. If we assume an individual autocorrelation of 0.8, a cluster autocorrelation of 0.5, an ICC of 0.01 and that 10 individuals were sampled from each agency, this gives us a design effect for clustering of 1.09 and a design effect for repeated measures of 0.09. Using a 5% significance level we will have more than 80% power to detect an average difference in SEER scores of 1.5 if the standard deviation is 6.

**Reference**

Hooper R, Teerenstra S, de Hoop E and Eldridge S. Sample size calculation for stepped wedge and other longitudinal cluster randomised trials. Statistics in Medicine, 2016, 35 4718–4728.
